# A review for non-antibiotic treatment of *Helicobacter pylori*: new insight

**DOI:** 10.3389/fmicb.2024.1379209

**Published:** 2024-05-07

**Authors:** Neda Shadvar, Sousan Akrami, Seyyed-Mohammad-Amin Mousavi Sagharchi, Rafee Habib Askandar, Alireza Merati, Masoomeh Aghayari, Nikki Kaviani, Hamed Afkhami, Mojtaba Kashfi

**Affiliations:** ^1^Department of Microbiology and Parasitology, School of Medicine, Bushehr University of Medical Sciences, Bushehr, Iran; ^2^Student Research Committee, Bushehr University of Medical Sciences, Bushehr, Iran; ^3^The Persian Gulf Tropical Medicine Research Center, The Persian Gulf Biomedical Sciences Research Institute, Bushehr University of Medical Sciences, Bushehr, Iran; ^4^Department of Microbiology, School of Medicine, Tehran University of Medical Sciences, Tehran, Iran; ^5^Department of Microbiology, College of Basic Sciences, Shahr-e Qods Branch, Islamic Azad University, Tehran, Iran; ^6^Research Center, Sulaimani Polytechnic University, Sulaymaniyah, Iraq; ^7^Department of Psychology and Educational Sciences, Payame Noor University, Tehran, Iran; ^8^Department of Microbiology, Faculty of Sciences, Urmia Branch, Islamic Azad University, Urmia, Iran; ^9^School of Medicine, Kermanshah University of Medical Sciences, Kermanshah, Iran; ^10^Nervous System Stem Cells Research Center, Semnan University of Medical Sciences, Semnan, Iran; ^11^Department of Medical Microbiology, Faculty of Medicine, Shahed University, Tehran, Iran; ^12^Fellowship in Clinical Laboratory Sciences, Mashhad University of Medical Sciences, Mashhad, Iran

**Keywords:** *Helicobacter pylori* (*H. pylori*), probiotic therapy, quadruple therapy, triple therapy, antibiotic

## Abstract

Gastric ulcers and gastric cancer are brought on by the *Helicobacter pylori* bacteria, which colonizes under the stomach mucous membrane. Different medication regimens are used to remove it, but the illness returns and becomes more resistant, which lowers the treatment rates. Additionally, this bacterium now exhibits a skyrocketing level of multi-drug resistance, necessitating recurrent therapeutic treatments. The negative effects of synthetic medications in comparison to conventional therapies are another significant factor in favor of non-pharmacological therapy. The most significant side effects of popular anti-gastric ulcer medications include nausea, vomiting, and diarrhea. Stomach ulcers have previously been treated with herbal remedies and complementary treatments like probiotics. When probiotics are ingested, the host experiences several advantages that may be brought about by altering the bacterial flora in the digestive system. Additionally, stronger-acting chemical compounds and plant extracts can be employed to treat patients. In this article, we look at the substances and medications that are utilized in place of synthetic stomach ulcer-curing treatments.

## Graphical abstract

Non-antibiotic treatment for *Helicobacter pylori*
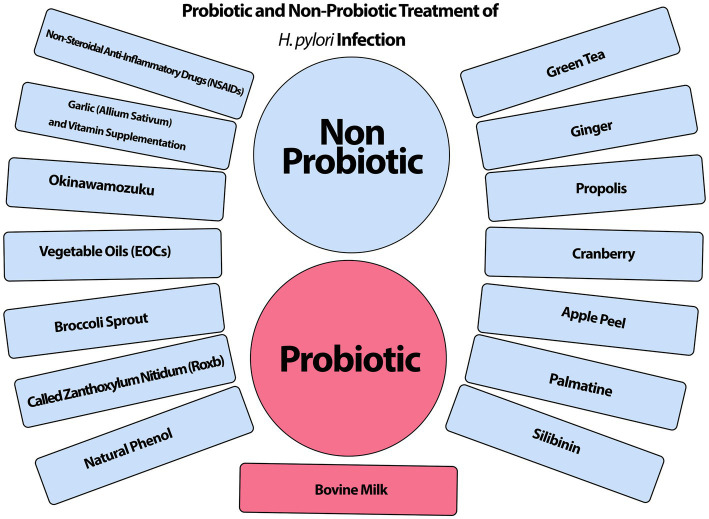
.

## Introduction

*Helicobacter pylori* (*H. pylori*) is a gram-negative bacterium, with a curved shape, flagellated. Several virulence factors are known in this bacterium that are involved in the process of pathogenesis, resistance to acid, drug resistance, and damage to the host (Ansari and Yamaoka, 2020; Sukri et al., 2020; Kolinjivadi et al., 2022). Flagella greatly increases the penetration power of bacteria into the submucosa of the stomach, which is important in bacterial pathogenesis (Baj et al., 2020; Khaledi et al., 2020; Afra et al., 2021). Other factors, such as the urease enzyme, are responsible for the survival of bacteria in the acidic conditions of the host’s stomach (Yang et al., 2023). Two other virulence factors include *cagA* and *vacA* are closely related to the occurrence of gastric cancer. Cytotoxin-Associated Gene A (CagA) is found in strains with high virulence that EPIYA (glutamic acid-proline-isoleucine-tyrosine-alanine) region in CagA, plays a significant role (Noiri and Nagata, 2023; Wang et al., 2023). VacA is a multifunctional toxin that is responsible for eliciting multiple effects on host cells (Salvatori et al., 2023). *Helicobacter pylori* is known to cause asymptomatic gastritis to chronic gastritis which, if left untreated, may lead to stomach ulcers, MALT (mucosa-associated lymphoid tissue lymphoma), and stomach cancer (Salama, 2020; Aumpan et al., 2023; Messina et al., 2023). *Helicobacter pylori* infection is thought to be acquired during early childhood by oral-oral or fecal-oral transmission channels and typically lasts a lifetime. Spontaneous clearance without encountering antibiotics is rare (Engelsberger et al., 2024). This bacterium is responsible for infecting approximately 4.4 billion people on Earth and 9% of all cancer-related deaths are associated with *H. pylori* (Savoldi et al., 2018). About 15%–25% of individuals in developed countries and up to 70%–90% of people in developing countries are infected with this bacterium (Fang et al., 2019; Abo-Amer et al., 2020). In some parts of the world, the prevalence of this disease and recrudescence is very high. Moreover, in parts of South America and China, and Eastern European about 80% or more have been documented (Choi et al., 2019; Qureshi et al., 2019; Sun and Zhang, 2019). The conventional therapy for *H. pylori* infection is proton pump inhibitors (PPIs) coupled with two antibiotics and bismuth (Savoldi et al., 2018). However, the development of antibiotic resistance has grown in recent years, resulting in a drop in *H. pylori* eradication rates. Furthermore, antibiotics may cause negative effects on the human gastrointestinal tract, such as diarrhea, anorexia, emesis, abdominal distension, and discomfort (Poonyam et al., 2019). As a result, there is a rising need for alternate medications to manage *H. pylori* infection successfully. Plant extract and probiotic-based foods are becoming increasingly popular. These innovative treatments are effective against *H. pylori* infection. This study includes a thorough assessment of the global prevalence, transmission pathways, and pathophysiology of *H. pylori* infection, as well as an overview of phytotherapy, probiotics, and new therapeutics, including their potential actions against *H. pylori*.

## Methods

The data and literature were searched in the databases SID, Irandoc, Magiran, Google Scholar, Web of Science, PubMed, and Scopus using the keywords *H. pylori*, alternative therapy, and non-pharmacological treatment from 2003 to 2023. This collection yielded various non-antibiotic therapeutic options, including probiotic therapy and non-probiotic product therapy.

## Routine treatment of *Helicobacter pylori*

Antimicrobial susceptibility testing (AST) and antibiotic resistance associated with geographical region should be taken into consideration when choosing the best regimen. In certain nations, a treatment regimen based on recommendations and the use of multiple medications (hybrid therapy) may be carried out concurrently for 10–14 days or more (Graham, 2015). In the past, triple therapy by combination of two antibiotics including clarithromycin and amoxicillin and plus one proton pump inhibitor (Di Fermo et al., 2020) such as omeprazole was considered the acceptable first-line regimen (Abadi, 2017; Chey et al., 2017; Malfertheiner et al., 2022). Second-line antibiotics were utilized because bacteria showed significant levels of resistance to first-line medications (Thung et al., 2016). Quadruple therapy by bismuth quadruple therapy (BQT), metronidazole, tetracycline, and omeprazole in some countries like the United States of America and Europe is suggested (Poonyam et al., 2019; Zama et al., 2020).

## Treatment failure

Patients frequently experience the need to repeat treatments, or complications from the disease reappear months or years after treatment. *Helicobacter pylori* can overcome environmentally stressed conditions, such as sub-inhibitory drug concentrations or a non-permissive atmosphere, by entering the viable but nonculturable (VBNC) state, in which the microorganism modifies its morphology from a spiral to a coccoid (spherical) form with a loss of cultivability (Cellini, 2014). Chaput et al. (2006) revealed that *H. pylori* coccoid cells can avoid detection by the immune system due to a considerable change of the cell wall peptidoglycan, which has no IL-8 stimulatory action in gastric epithelial cells. Thus, in the VBNC condition, *H. pylori* may be able to evade or regulate the host response, allowing it to survive in the human stomach. Wang and Wang (2004) created a coccoid *H. pylori* population by exposing these cells to a sub-inhibitory dosage of antibiotics, after which the target segment of the *cagA* gene was amplified, cloned into a plasmid, and transformed in *Escherichia coli*. The scientists used sequence analysis to show that coccoid *H. pylori* carried a full *cagA* gene with 99.7% similarity to the reported original sequence of vegetative forms of *H. pylori*, confirming conjecture regarding the pathogenicity of these cells. These considerations clearly emphasize that the morphological fickleness of *H. pylori* occurs in reaction to external stimuli entering the VBNC stage, and represents, over the lifespan of the microorganism, a potent response to promote bacterial “fitness” and species preservation. This critical survival strategy is reinforced when bacterial cells arrange themselves into microbial communities, generating a kind of “free multicellularity” and forming biofilm (Bessa et al., 2013). *Helicobacter pylori* is well recognized for its ability to create biofilm both outside and within the human host, which presumably provides greater protection under stressful situations. Bessa et al. (2013) found that sub-minimal Inhibitory Concentration (MIC) levels of amoxicillin and clarithromycin might enhance biofilm biomass. The influence of sub-MIC drugs on *H. pylori* biofilm-forming capability may have clinical implications, as during any antibiotic treatment focused on a specific infection, *H. pylori* bacteria can be exposed to sub-MICs of antibiotics, which can stimulate the switching from planktonic to sessile cells forming biofilm, resulting in recalcitrance to antibiotic treatment and thus preventing eradication. According to a recent thorough meta-analysis of 132 research (53,934 person-years) from 45 nations or regions, the global yearly reinfection rate of *H. pylori* was 3.1%, which has been generally consistent over the previous three decades but varied across different locations (Hu et al., 2017). One of the most important causes of treatment failure is multiple resistance to common drugs, which may repeat the course of treatment several times or use alternative drugs (Shah et al., 2021; Wallner et al., 2022). Some studies worldwide have shown that prevalence primary multidrug-resistant (MDR) *H. pylori* is increasing (Karbalaei et al., 2022). It has been reported that metronidazole resistance grew by nearly 50% in China over 14 years, from 2000 to 2014. Furthermore, during the same period in China, the clarithromycin resistance rate jumped from 14.8% to 52.6% (Thung et al., 2016). In this century, antibiotic resistance to metronidazole, amoxicillin, and clarithromycin has increased significantly (Adachi et al., 2023; Rokkas and Graham, 2023). Clarithromycin has emerged as the foundation for *H. pylori* treatment in combination therapy because of its minor effect on stomach acidity, low threshold inhibitory concentration, and reasonably excellent mucosal diffusion (Marques et al., 2020). In developing nations, clarithromycin resistance and the frequency of re-infection contribute to the high global incidence of *H. pylori* infection and consequent inflammatory and neoplastic diseases (Alarcón-Millán et al., 2016). In most European nations and the rest of the world, the prevalence of clarithromycin resistance has reached 20% (Morilla et al., 2019). The discrepancy in clarithromycin resistance rates among the included studies might be attributed to a variety of factors, including variances in antibiotic prescription rates across geographic areas, the use of different breakpoints or erroneous criteria in conducted research, and the introduction of MDR strains. In a comprehensive review of the literature in Iran, *H. pylori* resistance to several antibiotics, including metronidazole, clarithromycin, amoxicillin, tetracycline, ciprofloxacin, levofloxacin, furazolidone, was 61.6%, 22.4%, 16.0%, 12.2%, 21.0%, 5.3%, and 21.6%, respectively (Khademi et al., 2015). *Helicobacter pylori* resistance to metronidazole is far higher in Iran than in industrialized nations. *Helicobacter pylori* resistance to fluoroquinolones (i.e., ciprofloxacin, moxifloxacin, trovafloxacin, and levofloxacin), nitrofurans (i.e., furazolidone), and rifamycins (i.e., rifabutin) is 0% to 20%, 0% to 5%, and 0% to 2%, respectively, in different geographic locations of the world (Hosseini et al., 2012). Antibiotic resistance is minimal in Middle Eastern nations (Khademi et al., 2015). In recent years, furazolidone has been considered a good alternative to metronidazole and tetracycline in quadruple therapy for eradication of *H. pylori* in Iran, but at the moment, the resistance of *H. pylori* to furazolidone is detected in 21.6% of cases, which is higher than the global average (Khademi et al., 2015). Resistance rates for rifabutin were 0.0, 6.4% for amoxicillin, 17.4% for clarithromycin, and 43.6% for metronidazole in the largest clinical trial of *H. pylori* antimicrobial susceptibility in more than 15 years, conducted in 20 states in the United States. The dual resistance rate to metronidazole and clarithromycin was 10.5% (Mestrovic et al., 2020). As treating *H. pylori* is never an emergency, choosing a regimen based on local susceptibility patterns or, ideally, the susceptibility profile of an individual’s infecting strain would be a better approach, as it would prevent patient exposure to antimicrobials that are ineffective against a resistant *H. pylori* strain. According to the research, it appears that in addition to antibiotic therapy, it is preferable to employ additional therapies such as probiotics, plant extracts, and any effective combination against *H. pylori*.

## Alternative therapies

### Probiotics

Probiotics are living bacteria that provide several advantages to the body and are used orally to treat gastrointestinal problems (Losurdo et al., 2018). Probiotic preparations are available in a variety of formats, including capsules, solutions, powders, and probiotic foods (Kiepś and Dembczyński, 2022). Probiotics have a lot of potential as antibiotic replacements. Following the investigation by Yoon et al. (2019), a reduction in *H. pylori* density and histologic inflammation improvement could be detected in individuals treated with fermented milk containing *Lactobacillus paracasei* HP7 and herbal extract (*Glycyrrhiza glabra*). In a randomized double-blind placebo-controlled clinical trial, the decrease in the mean *H. pylori* stool antigen titer showed a significant difference between *Saccharomyces boulardii* and the control group, indicating that *S. boulardii* could positively reduce *H. pylori* colonization in the human gastrointestinal system, but it is incapable of eradication as monotherapy (Namkin et al., 2016). As a result, probiotic monotherapy cannot be utilized to treat *H. pylori*, even though it may suppress bacterial growth. Probiotics have a good influence on digestive health, reinforce the mucosal barrier on mucus against pathogens, improve immune system, and anti-pathogen action via antimicrobial factor release, aggregation and co-aggregation by attachment to pathogens (Agraib et al., 2021; Keikha and Karbalaei, 2021; Daelemans et al., 2022; Du et al., 2022). In addition, probiotics are used in the treatment of gastrointestinal cancer (Zhang et al., 2021). Clinical experiments have shown that probiotic lactic acid-producing bacteria are a simple, safe, and effective way to prevent cancer patients against radiation-induced diarrhea. Patients with colorectal cancer were randomly assigned to take *L. rhamnosus* GG supplements and fiber throughout treatment. Patients who took *Lactobacillus* experienced less grade 3 or 4 diarrhea, reported less abdominal discomfort, required less hospitalization, and had fewer chemotherapy dosage decreases owing to bowel damage. There was no *Lactobacillus*-related harm found (Delia et al., 2007; Österlund et al., 2007). The study concludes that *Lactobacillus* GG supplementation is well tolerated and may minimize the incidence of severe diarrhea and abdominal pain associated with 5-FU-based chemotherapy. Generally, probiotics have been added to dairy products such as yogurt and other fermented products.

**Figure 1:**
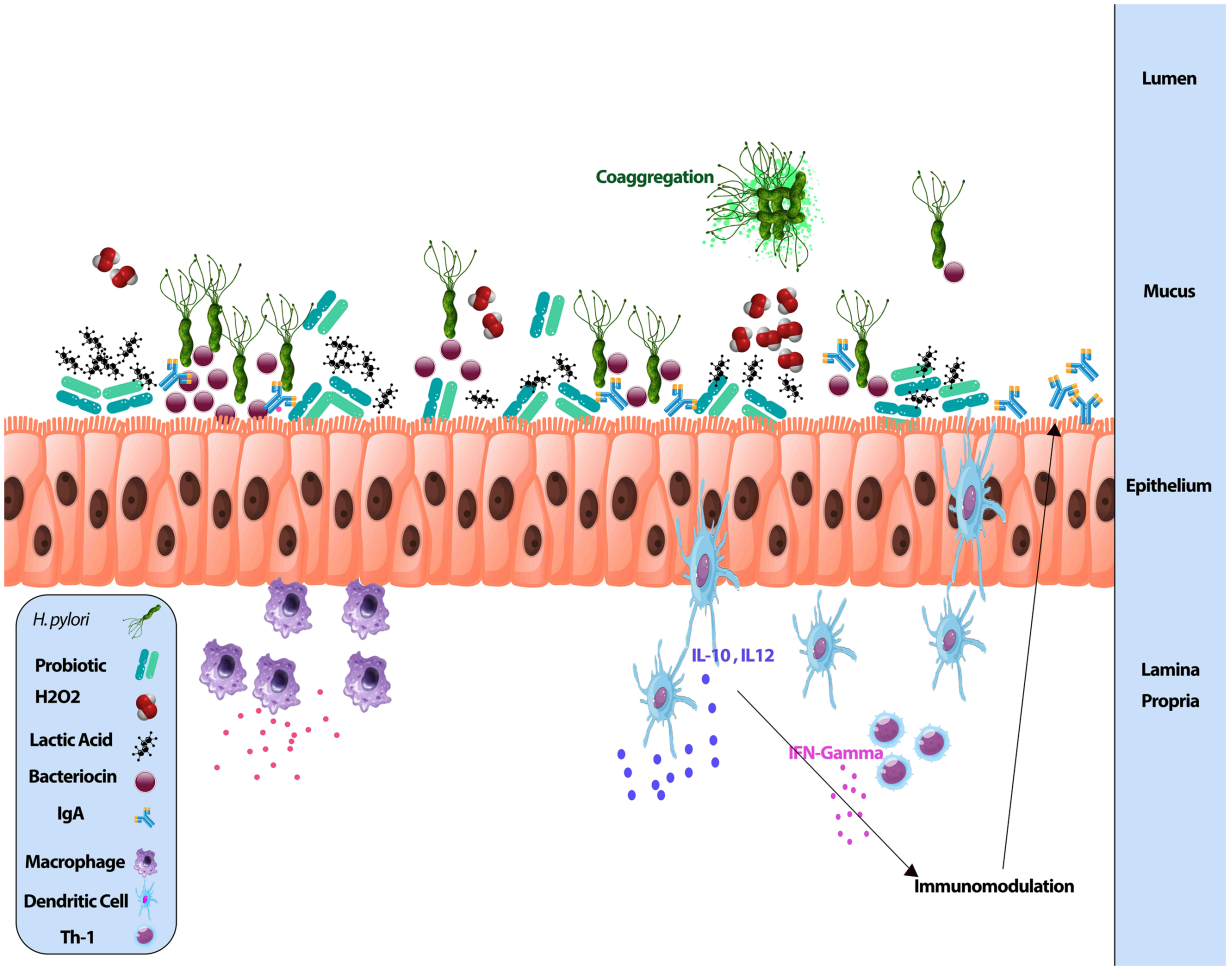
Probiotics in the digestive tract.

*Lactobacillus and Bifidobacterium are the two most common species found in probiotic products such as yogurt* (Xiong, 2018; Ye et al., 2020). In the past, the positive effects of dairy products such as yogurt on digestion were proven (Whiteside et al., 2021; Zhao et al., 2022). Other species accessible on the market include *Bacillus* spp., *Enterococci*, *E. coli*, *Weissella* spp., and *Saccharomyces*. Classical probiotic strains containing *Lactobacilli* and *Bifidobacterium* have been granted Generally Regarded as Safe (GRAS) status in the United States and are eligible for EFSA (European Food Safety Authority) safety certification. Microbial strains in multi-species probiotics include *Bifidobacterium breve, Bifidobacterium infantis, Bifidobacterium longum, Eubacterium faecium, Lactobacillus acidophilus, Lactiplantibacillus plantarum, Lacticaseibacillus casei*, and *Streptococcus thermophilus*. Researchers discovered that probiotic mixes including many strains outperformed single-strain probiotics in terms of pathogenic growth inhibition (Singh et al., 2022). These bacteria may colonize the human gastrointestinal tract and compete with *H. pylori* for attachment to the host’s mucous membrane (Espinoza et al., 2018). *Lactobacillus* secretes chemicals that inhibit *H. pylori* urease activity and colonization. *Bifidobacterium* reduces *H. pylori* adherence to intestinal mucus by site competition, and it has been found to lower IL 8 levels *in vivo* and *in vitro H. pylori* activity (Koca et al., 2021). In the Maastricht V Consensus Report, it has been agreed that some probiotic strains may have favorable effects on *H. pylori* elimination (Malfertheiner et al., 2017). Given these findings, it is plausible to expect that utilizing a combination of probiotics that act on *H. pylori* through diverse routes might result in a synergistic impact. Probiotics used in combination with antibiotics have been demonstrated in studies to improve Helicobacter eradication. When double probiotics were added to the medication, the number of significant adverse effects decreased by 11.2%. *Helicobacter* eradication was accomplished in 68% of patients in the control group and 89% of patients in the trial group following therapy completion. When double probiotics were added to the therapy, the eradication success rate improved by 21% (Koca et al., 2021). While *H. pylori* disturbs the normal flora in the stomach microbiota, antibiotic therapy causes a long-term decline in normal strains in the intestinal flora. Probiotics protect the intra-stomach microbiota against the detrimental effects of *H. pylori* and restore the degraded intestinal flora. *Helicobacter pylori* can cause an inflammatory response by altering the acid–base balance of the digestive system and, as a result, the gut microenvironment (Chen et al., 2018; Yoon et al., 2019). Hence probiotic supplementation may be beneficial in the treatment of inflammatory and infectious diseases (Chen et al., 2018). Figure 1 shows the use of probiotics in the digestive tract schematically. Epithelial commensals and probiotics promote barrier integrity. Probiotics play a significant function in maintaining immunological balance in the gastrointestinal system by interacting directly with immune cells. Probiotic strains increase IgA synthesis and secretion by altering the cytokine milieu in the gut mucosa. Also, bacteriocins are antimicrobial peptides generated by the majority of bacterial species. They have been thought to contribute to probiotic efficacy by assisting with colonization, direct pathogen eradication, and acting as signaling molecules to other bacteria or the host immune system.

Probiotics have few adverse effects associated with bacterial-host interactions. Individuals suffering from immunodeficiency, heart valve disease, short bowel syndrome, or preterm newborns are at risk of severe responses and should avoid using these items (Doron and Snydman, 2015; Singhi and Kumar, 2016). Probiotic therapy is a first medication for *H. pylori* treatment in order to optimize eradication regimens and reduce antibiotic adverse effects (Chey et al., 2017). As *H. pylori* has long been seen as a difficult-to-treat disease, due mostly to developed resistance to routinely used antibiotics, there is a rising interest in using probiotics in combination with antibiotic regimens to remove the bacteria. Certain *Lactobacillus* species produce antibacterial chemicals similar to the bacteriocin class. Bacteriocins are proteinaceous toxins with anti-*H. pylori* properties. They are tiny, dialyzable peptide structures that possess antibacterial properties. The antibacterial activity of the bacteriocins differed depending on the *H. pylori* strain and the kind of bacteriocin generated by *Lactobacillus* sp. Some bacteriocins exhibit more antibacterial action against *H. pylori* strains than others (Kim et al., 2003). Based on clinical trials with probiotics against *H. pylori*, probiotics cannot be considered an alternative to anti-*H. pylori* treatment; however, their use together with standard anti-*H. pylori* treatment may improve *H. pylori* treatment by increasing eradication rates while decreasing the adverse effects of current medications. *Lactobacillus acidophilus* was a little better choice for 7- and 14-day triple therapy, whereas *S. boulardii* was better suited to 10-day triple therapy. When combined with *H. pylori* eradication medication, most probiotic regimens proved to be more successful than placebos. Additionally, probiotics are indicated to enhance triple treatment in kids. To augment triple treatment, *L. casei* was discovered as the best for *H. pylori* eradication rates, and multi-strain of *L. acidophilus* and *L. rhamnosus* for overall adverse effects (Feng et al., 2017; Wang et al., 2017). While it may be generally acknowledged that probiotics may help in the eradication of *H. pylori* and reduce adverse effects of standard therapy, certain probiotic bacteria species can also benefit pharmacotherapy. The growing prevalence of antibiotic resistance and the decrease in patient compliance with traditional treatment further illustrate the need for alternative therapies. Fakhry et al. (2023) found that patients who received probiotics (*Lactobacillus* and *Bifidobacterium*) in addition to regular medicine (triple treatment) had a higher percentage of *H. pylori* eradication than those who received only conventional therapy. Adding probiotics reduced diarrhea as a side effect of antibiotic treatment. Probiotics may help to repair gut dysbiosis, as demonstrated by stool PCR for Lactobacilli and Bifidobacteria before and following drugs. Probiotic supplements may assist to reestablish a favorable gut microbial composition, especially following eradication treatment.

The composition of the human gastrointestinal microbiota tract has been extensively examined, and various studies have been undertaken to explain the links between microbiota diversity in the human gastrointestinal tract and its impact on health and illness. Differences in gastric bacterial community topologies may govern diverse pathways, affecting stomach physiology and leading to varied *H. pylori* infection responses (Ge et al., 2018). Commensal gastric microorganisms or their metabolites impact *H. pylori*’s capacity to colonize the stomach and its pathogenic and carcinogenic potential via influencing host immune responses (Espinoza et al., 2018). Non-*H. pylori* bacteria may survive as an antigenic stimulation or form a partnership with *H. pylori* to increase eventual inflammation (Rook et al., 2017). Studies discovered that stomach microbial populations considerably enhanced their alpha-linolenic acid and arachidonic acid metabolism. Therefore, Zheng et al. (2021) hypothesized that the balance between Treg and Th17 cells might be biased toward Treg cells, which is beneficial to bacterial persistence, and the gastric microbiota might generate short-chain fatty acids and small molecules to modulate mucosal Treg responses in *H. pylori*-infected patients. Furthermore, Satoh-Takayama et al. (2020) demonstrated that ILC2s, controlled by local commensal populations through IL-7 and IL-33 upregulation, are the main ILC subset in the stomach and protect against *H. pylori* infection via B-cell activation and IgA production.

Probiotics can change the structure of the stomach microbiota. Probiotic therapy increased the amount of beneficial short-chain fatty acid (SCFA) generating bacteria, including *Bacteroides*, *Alloprevotella*, and *Oscellibacter*, in the stomachs of *H. pylori*-infected mice (He et al., 2022). Sodium butyrate, a typical SCFA, suppressed *H. pylori* growth, CagA and VacA expression, as well as the host NF-κB pathway by lowering toll-like receptor expression in host cells, resulting in decreased TNF-α and IL-8 production (Huang et al., 2021). However, another bacterial metabolite, trimethylamine N-oxide (TMAO), boosted *H. pylori* viability and virulence and aggravated *H. pylori*-induced inflammation (Wu et al., 2020). The synergistic effects of *H. pylori* and TMAO boosted inflammation-related gene expression, including IL-6, CXCL1, CXCL2, FOS, and complement C3 in the gastric epithelium (Wu et al., 2017). Trimethylamine (TMA) is the TMAO precursor. Firmicutes (e.g., *Staphylococcus*) are the primary producers, while Bacteroidetes generate it infrequently (Fennema et al., 2016). Overall, probiotics may raise the proportion of helpful metabolite-producing bacteria and/or lower the number of detrimental metabolite-producing bacteria (Zhang et al., 2023). Probiotics can help repair the stomach dysbiosis produced by eradication therapy, however, young adults infected with *H. pylori* may not need to take probiotics alone (Yuan et al., 2021).

### Synbiotics

Synbiotics are a mix of probiotics (“live microorganisms that, when administered in adequate amounts, confer a health benefit on the host”) and prebiotics (“indigestible foods that lead to enhancement of probiotic bacteria colonization in the gut”) that can operate synergistically. Several studies have found that some probiotic lactobacilli exhibit anti-*H. pylori* action and prevent *H. pylori* colonization in gastric cell lines (Pourmasoumi et al., 2019). A randomized, open-labeled trial examined the efficacy of a synbiotic combination (containing lactobacillus, enterococcus, and bifidobacterium) in clarithromycin-based triple eradication treatment for *H. pylori*. The data showed no significant difference between the two groups. However, the level of adverse effects and rates of eradication differed considerably between groups. The eradication rate in the synbiotic group was 88.4%, compared to 68.8% in the control group. The inclusion of synbiotics in triple treatment reduces the incidence of antibiotic-related adverse effects. It also improves *H. pylori* eradication in clarithromycin-based triple treatment (Sahin et al., 2013). A meta-analysis of six randomized controlled studies revealed that synbiotics could increase *H. pylori* eradication rates while reducing side effects (Pourmasoumi et al., 2019).

### Postbiotics

A novel biotherapeutic technique includes using microbial bioactive compounds (postbiotics) that display optimal compatibility and intimate interaction with the host’s immune system (Hosseini et al., 2023). Postbiotics can also compete with pathogens for adhesion sites if their adhesions (such as fimbriae and lectins) are still functional after pretreatment. *Lactobacillus acidophilus* in lyophilized and inactivated form significantly boosts *H. pylori* eradication rates when added to a standard anti-*H. pylori* regimen, owing to its strong adhesion to human intestine absorptive and mucous-secreting cells. Given its safety and high patient compliance, it is a simple addition to traditional anti-*H. pylori* antibiotic treatments (Ma et al., 2023). There is scant evidence that postbiotics are effective in treating human diseases. We uncovered two separate clinical studies using postbiotics for *H. pylori* infection. Canducci et al. (2000) found that treating patients with *H. pylori* with clarithromycin, rabeprazole, and amoxicillin with inactivated *Lactobacillus acidophilus* increased the rate of eradication. Yang et al. found that adding nonviable *L. reuteri* to triple therapy (esomeprazole, amoxicillin, and clarithromycin) did not increase the rate of *H. pylori* eradication, but did help to establish a beneficial microbial profile and reduce the occurrences of abdominal distention and diarrhea (Hamzavi and Bashiri, 2023).

### Non-probiotic products

The positive benefits of probiotics are well recognized, but other components such as plant extracts, oils, and derivatives of some natural chemicals can help reduce pathogenesis and cancer growth.

**Figure 2:**
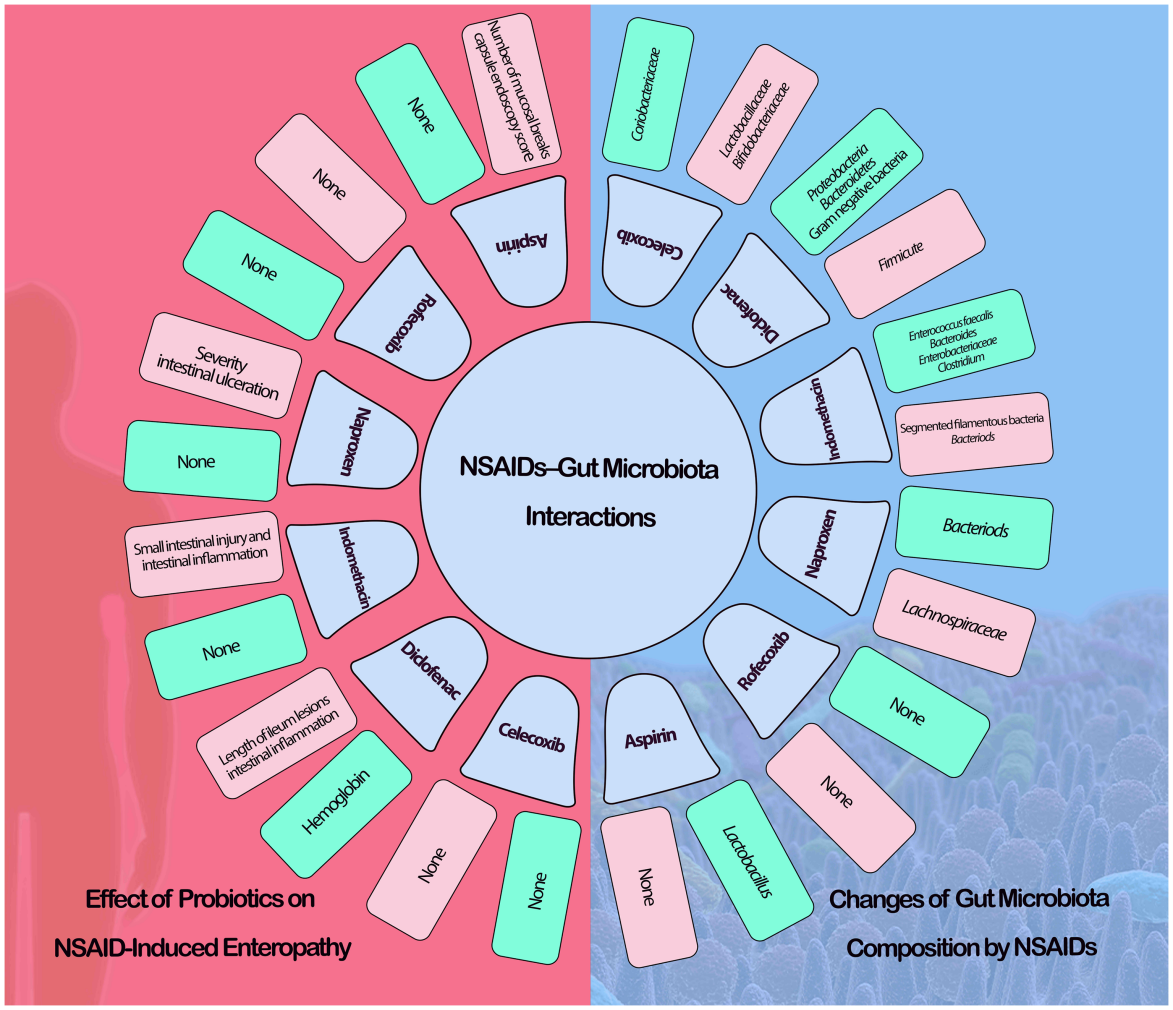
NSAIDs-gut microbiota interactions (green: increase, red: decrease).

Nonsteroidal anti-inflammatory drugs (NSAIDs) such as acetaminophen, ibuprofen, diclofenac, and acetyl Salic acid are among the most commonly used antibacterial agents in the world (Zimmermann and Curtis, 2017). Salicylic acid (SAL) has been proven in studies to reduce the growth of *H. pylori* and *Klebsiella pneumonia* (Reshetnyak et al., 2019). Other NSAIDs can have an effect on bacteria; for example, ibuprofen can reduce the binding of *Escherichia coli* to intestinal epithelial cells or uroepithelial cells (Whiteside et al., 2019; Figure 2). Another study published in 2018 found that diclofenac loaded with chitosan nanoparticles may inhibit the growth of *S. aureus* and *B. subtilis* (Alqahtani et al., 2019). Resveratrol (RSV) and its derivatives are a kind of natural phenol with anti-inflammatory and antibacterial properties (Vestergaard and Ingmer, 2019). RSV works by inhibiting the urease enzyme and, as a result, preventing the formation of an alkaline environment (Marini et al., 2019; Krzyżek et al., 2020). Several investigations have demonstrated that anti-helicobacter drugs such as levofloxacin have higher bacterial toxicity when combined with Resveratrol and its derivatives (Bouarab Chibane et al., 2019; Di Lodovico et al., 2019; Di Fermo et al., 2020). Polaprezinc (PZN) or zinc carnosine (L-carnosine) are zinc chelate compounds that protect the digestive mucosa (Mahmoud et al., 2022). This antioxidant compound is particularly efficient in protecting against *H. pylori* and promoting ulcer healing, and unlike other antibiotics, it does not cause drug resistance (Teng et al., 2020; Ibrahim et al., 2022; Mahmoud et al., 2022). PZN has been shown in several trials to be useful in reducing apoptosis and inflammation, healing skin and stomach wounds, and preserving tight junctions (Furihata et al., 2020; Ibrahim et al., 2022). Hence, this substance may be introduced later in the *H. pylori* treatment protocol (Kawahara et al., 2018; Mahmoud et al., 2022). Palmatine (Pal) is a herbal combination derived from Coptidis Rhizoma that may relieve or diminish chronic atrophic gastritis (CAG) caused by *H. pylori*. Pal most likely does this by suppressing inflammatory compounds including interleukin 8 (IL-8) and chemokine 16 (CXCL-16; Chen et al., 2020). Silibinin, derived from milk thistle seeds, possesses potent anti-Helicobacter and anti-gastric tumor cell properties (Bittencourt et al., 2020). This chemical affects Penicillin Binding Protein (PBP) and interferes with wall formation, resulting in changes in the bacterial structure. It also has anti-inflammatory properties by decreasing cytokine release by inhibiting macrophages activated by *H. pylori* (Bittencourt et al., 2020; Cho et al., 2021). Vergara et al. (2005) found that eradicating *H. pylori* was successful in NSAID-naive users but not in chronic users.

Some foods, edible plants, and natural dairy products exhibit antibacterial characteristics *in vitro* or *in vivo*, including *H. pylori*. Most of these drugs’ effective composition is known (Takeuchi et al., 2014). The most well-known compounds that are effective against bacteria that cause stomach ulcers include bovine milk (by Lactoferrin), ginger (6-shogaol or phenolic acids), broccoli sprout (sulforaphane), green tea (Catechin compounds), and garlic (Allicin, diallyl sulfur components; Cellini et al., 1996; Sachdeva and Nagpal, 2009; Sachdeva et al., 2014; Omar et al., 2020; Zhao et al., 2022). *In vitro* tests showed that bovine lactoferrin (bLF) had a strong antibacterial impact on resistant *H. pylori* and a synergistic antibacterial effect when paired with clarithromycin. Furthermore, *in vivo* investigations indicated that bLF might enhance the degree of gastric mucosa damage and minimize the area of the stomach ulcer (Wang et al., 2023). According to the findings of researchers, the methanolic extract of ginger, which contains gingerol, zingiberene, and thymol as major compounds, has potential antibacterial and antibiofilm properties against various multidrug-resistant clinical isolates of *H. pylori*, as well as anti-inflammatory activity. The results mentioned demonstrate a considerable improvement of gentamicin effectiveness against multidrug-resistant *H. pylori* when combined with methanolic ginger extract (Elbestawy et al., 2023). To find out if regular broccoli sprout consumption had a similar effect in humans, researchers enrolled 50 *H. pylori*-infected individuals in a clinical trial. When measurements obtained at baseline were compared with those taken after one or 2 months of intervention, significant decreases in the number of bacteria were evident in individuals eating broccoli sprouts (Tuma, 2006). Yanagawa et al. (2003) demonstrated that epigallocatechin-3-gallate (from green tea) enhanced the antibacterial activity of an antibiotic treatment, with excellent efficacy against *H. pylori* growth *in vitro*. García et al. (2023) found that solvent-free garlic extracts containing ethanol and acetone suppressed *H. pylori* development *in vitro* under simulated stomach PH conditions at human body temperature. These results imply potentially major medicinal uses of such extracts, which eliminate the usage of proton pump inhibitors during the treatment of *H. pylori* infections in human patients.

Bactericidal, antitoxin, and antibiofilm properties of vitamin and garlic supplements can protect those at risk of stomach cancer (Li et al., 2019; Bhatwalkar et al., 2021). Garlic contains chemicals that protect the genome structure by suppressing reactive oxygen and radical scavenging (Vega-Hissi et al., 2019). According to these findings, food and other consumable things should be used in combination with antibiotics (Takeuchi et al., 2014). Numerous plants and vegetable oils, such as Chinese chive, are being studied, although the mechanism of action is unknown (Guerra-Valle et al., 2022). Some research has revealed that essential oil components (EOCs) have anti-*H. pylori* potential, and their mode of action is through immobilized antimicrobials (Ruiz-Rico et al., 2020). Table 1 lists the most important chemicals found in foods and spices, as well as the known mechanisms involved in anti-*H. pylori* action.

**Table 1:** Anti-*Helicobacter pylori* chemicals found in foods.

Products	Foodstuff	Mechanism(s)	References
Probiotic	Bovine milk	Increasing the effectiveness of antibiotic therapy and inhibition attachment to the mucous layer of the gut	Spitsberg (2005) and Wan et al. (2022)
Postbiotics	*L. rhamnosus* GG gelatine capsules	Preventing cancer patients against radiation-induced diarrhea	Österlund et al. (2007)
	*Lactobacillus acidophilus* and *L. bulgaricus*, *in vitro*	Inhibiting *H. pylori* adherence to human gastric epithelial cell line	Song et al. (2020)
	Quadruple therapy supplemented with *Clostridium butyricum*	Relieving gastrointestinal symptoms by inducing alterations in gut microbiota and host immune responses in *H. pylori*-positive patients	Chen et al. (2018)
	Triple therapy and yogurt-containing probiotics supplementation	Less diarrhea as a side effect of antibiotic therapy	Fakhry et al. (2023)
	Capsule contained *L. acidophilus* strain LB	Greater rate of *H. pylori* eradication	Canducci et al. (2000)
	Adding nonviable *L. reuteri* to triple therapy	Decrease the abdominal distention and diarrhea	Yang et al. (2022)
Non-probiotic	Green tea	Disrupted cell membrane and inhibition of urease	Omar et al. (2020) and Habbash et al. (2022)
	Non-steroidal anti-inflammatory drugs (NSAIDs)	Acetaminophen, ibuprofen, diclofenac and salicylic acid have anti-inflammatory effects	Zimmermann and Curtis (2017)
	Garlic (*Allium sativum*) and vitamin supplementation	Inhibiting reactive oxygen and radical scavenging	Li et al. (2019), Vega-Hissi et al. (2019), and Bhatwalkar et al. (2021)
	Ginger	Blockage of Toll-like receptor 4 (TLR4) activation	Kashani et al. (2021) and Magdy et al. (2023)
	Okinawamozuku	Inhibition of *H. pylori* attachment to the mucous layer of the gut	Tomori et al. (2019) and Tomori et al. (2021)
	Propolis	Destruction of the bacterial membrane	Romero et al. (2019) and Song et al. (2020)
	Vegetable oils (EOCs)	Unknown	Ruiz-Rico et al. (2020) and Guerra-Valle et al. (2022)
	Cranberry	Inhibition of *H. pylori* attachment to the mucous layer of the gut	Howell (2020), Nikbazm et al. (2022), and Wang et al. (2023)
	Apple peel	Inhibition of urease activity	Yang et al. (2022) and Kumari et al. (n.d.)
	Broccoli sprout	Decreased activation of inflammatory cytokines such as TNF-α and IL-1β	Azizi-Soleiman and Zamanian (2020), Ullah et al. (2021), and Holman et al. (2022)
	Natural phenol	Polaprezinc (PZN) or zinc carnosine (L-carnosine) have preventing apoptosis and inflammation	Furihata et al. (2020) and Ibrahim et al. (2022)
	Called Zanthoxylum nitidum (Roxb)	Anti-inflammatory effects	Lu et al. (2020)
	Palmatine	Inhibiting interleukin 8 (IL-8) and chemokine 16 (CXCL-16)	Chen et al. (2020)
	Silibinin	Inhibition of wall synthesis by PBP inhibition	Bittencourt et al. (2020) and Salatin et al. (2022)

A compound called Zanthoxylum nitidum (Roxb) is used in China and in traditional medicine. This substance has anti-inflammatory effects for treating stomach ulcers (Lu et al., 2020).

## Conclusion

Failure to treat *H. pylori* infection can result in gastric cancer; also, the bacterium has a lot of resistance and the diversity of disorders caused by *H. pylori* will become more apparent with time, requiring the development of stronger or other treatments. The side effects of medications, as well as the problems that patients experience during or after therapy, motivate us to develop treatments with fewer negative effects. Treatment is complex and has several limits due to bacteria’s strong resistance to drugs. Much research in the field of herbal medicines and natural goods has been completed, indicating that therapy should be done concurrently with chemical treatment and natural and effective products. Combination therapy, as well as the use of probiotics and micronutrients such as vitamin C, vitamin B12, vitamin E, and iron in *H. pylori*-infected persons, appear to be useful in the eradication of *H. pylori*. Probiotics reduce adverse effects and increase antibiotic effectiveness, most likely because they mirror the function of the human microbiome. Postbiotics can also eradicate infections through competition for adhesion sites. *Lactobacillus acidophilus* in lyophilized and inactivated form significantly boosts *H. pylori* eradication rates when added to a routine anti-*H. pylori* eradication regimen, due to its powerful adherent capacity to human intestine absorptive and mucous-secreting cells. As a result, novel drugs and other treatments for the condition should be developed as quickly as possible.

## Author contributions

NS: Writing – original draft, Data curation. SA: Writing – original draft, Data curation SMA.MS: Writing – original draft, Data curation. RHA: Writing – review & editing. AM: Writing – review & editing. MA: Writing – review & editing. NK: Writing – review & editing. HA: Methodology, Project administration, Supervision, Writing – review & editing. MK: Methodology, Project administration, Supervision, Writing – review & editing, Data curation, Software, Writing – original draft.
